# Interaction of OKL38 and p53 in Regulating Mitochondrial Structure and Function

**DOI:** 10.1371/journal.pone.0043362

**Published:** 2012-08-17

**Authors:** Jing Hu, Hongjie Yao, Fei Gan, Anthony Tokarski, Yanming Wang

**Affiliations:** Department of Biochemistry and Molecular Biology, Center for Eukaryotic Gene Regulation, Pennsylvania State University, University Park, Pennsylvania, United States of America; University of Medicine and Dentistry of New Jersey, United States of America

## Abstract

The tumor suppressor p53 is a well-known transcription factor controlling the expression of its target genes involved in cell cycle and apoptosis. In addition, p53 also plays a direct proapoptotic role in mitochondria by regulating cytochrome *c* release. Recently, we identified a novel downstream target of p53, OKL38, which relocalizes from nucleus to mitochondria upon forced expression to induce apoptosis. However, the mechanism underlying OKL38 targeting to mitochondria and apoptosis induction remains unclear. Here, we found that OKL38 interacts with p53 to regulate mitochondria function. After DNA damage, OKL38 colocalizes with p53 to mitochondria in U2OS cells. Further, p53 and OKL38 are targeted to mitochondria in synergy: forced expression of OKL38 leads to p53 localization to mitochondria while the expression of a mitochondria enriched p53 polymorphic variant, p53^R72^, leads to OKL38 enrichment in mitochondria. Biochemical analyses found that OKL38 and p53 interact *in vivo* and *in vitro* via multiple domains. In cell biological assays, multiple regions of OKL38 mediate its mitochondria localization and induce mitochondria morphology changes. OKL38 induces formation of megamitochondria and increases cellular levels of reactive oxygen species. Furthermore, OKL38 induces cytochrome *c* release upon incubation with mitochondria. Taken together, our studies suggest that OKL38 regulates mitochondria morphology and functions during apoptosis together with p53.

## Introduction

In response to various stress signals, the tumor-suppressor p53 modulates the transcription of hundreds of genes, which in turn regulate many biological processes, such as cell growth and proliferation, genome integrity, apoptosis, autophagy, angiogenesis, and reproduction [1]–[4]. For example, induction of an inhibitor of the cyclin-dependent kinases, p21/WAF1/CIP1, leads to cell cycle block at the G1 phase [5], while induction of Bax [6] and NOXA [7] leads to apoptosis. The transcriptional activity of p53 is fine tuned by many p53 cofactors, which modify p53 as well as chromatin by post-translational modifications, including phosphorylation, acetylation, and methylation [8]–[12].

In addition to its well-known function in transcription, a flurry of literature suggests that p53 directly regulates apoptosis in mitochondria [13]–[16]. Mitochondria play an important role in cell growth and apoptosis as well as in organism health and disease [17]–[19]. p53 interacts with multiple factors (e.g., Bcl-x_L_ and Bak), which regulate mitochondria membrane permeability and induce cytochrome *c* release and apoptosis [13]–[16]. The accumulation of p53 in mitochondria has been detected in cells after gamma irradiation [20] or oxidative stress [21]. Notably, a common p53 polymorphic allele, p53^R72^ (carrying an Arg instead of Pro at residue 72), has greater ability in mitochondria localization and apoptosis induction compared with the p53^P72^ allele [22]. It has been implicated that the increased binding of p53^R72^ with the proapoptotic protein Bak may contribute to the increased ability of p53^R72^ in apoptosis induction [14].

Recently, we have identified a novel p53 target gene, *OKL38*, which is implicated in apoptosis by inducing cytochrome *c* release [23]. We and others found that OKL38 is inducible by DNA damaging reagents or oxidative stress [23]–[25], suggesting that the *OKL38* gene expression is modulated by distinct stress signals. Forced expression of OKL38 has been correlated with cell growth inhibition and apoptosis induction in multiple cell types [23], [26], [27]. Conversely, the loss or decreased expression of OKL38 protein has been detected in a high percentage of liver and kidney tumors [28], [29]. Thus, OKL38 likely plays a critical role in multiple tissues to guard against tumorigenesis. However, the molecular mechanisms underlying OKL38 function during cell growth, proliferation, and apoptosis remain largely unclear.

Here, we show that OKL38 and p53 assist each other in mitochondrial targeting to regulate mitochondrial morphology and function. Protein-protein interaction studies found that OKL38 and p53 interact *in vitro* and *in vivo*. We further showed that multiple domains of OKL38 interact with p53 and impact on mitochondria morphology. Moreover, we showed that OKL38 elevates cellular levels of reactive oxygen species (ROS) that are important regulators of mitochondrial functions, and induces cytochrome *c* release from mitochondria in biochemical assays. Taken together, our data suggest that OKL38 works with p53 in regulating mitochondria morphology and function.

## Results

### Colocalization of OKL38 and p53 in Mitochondria

**Figure 1:**
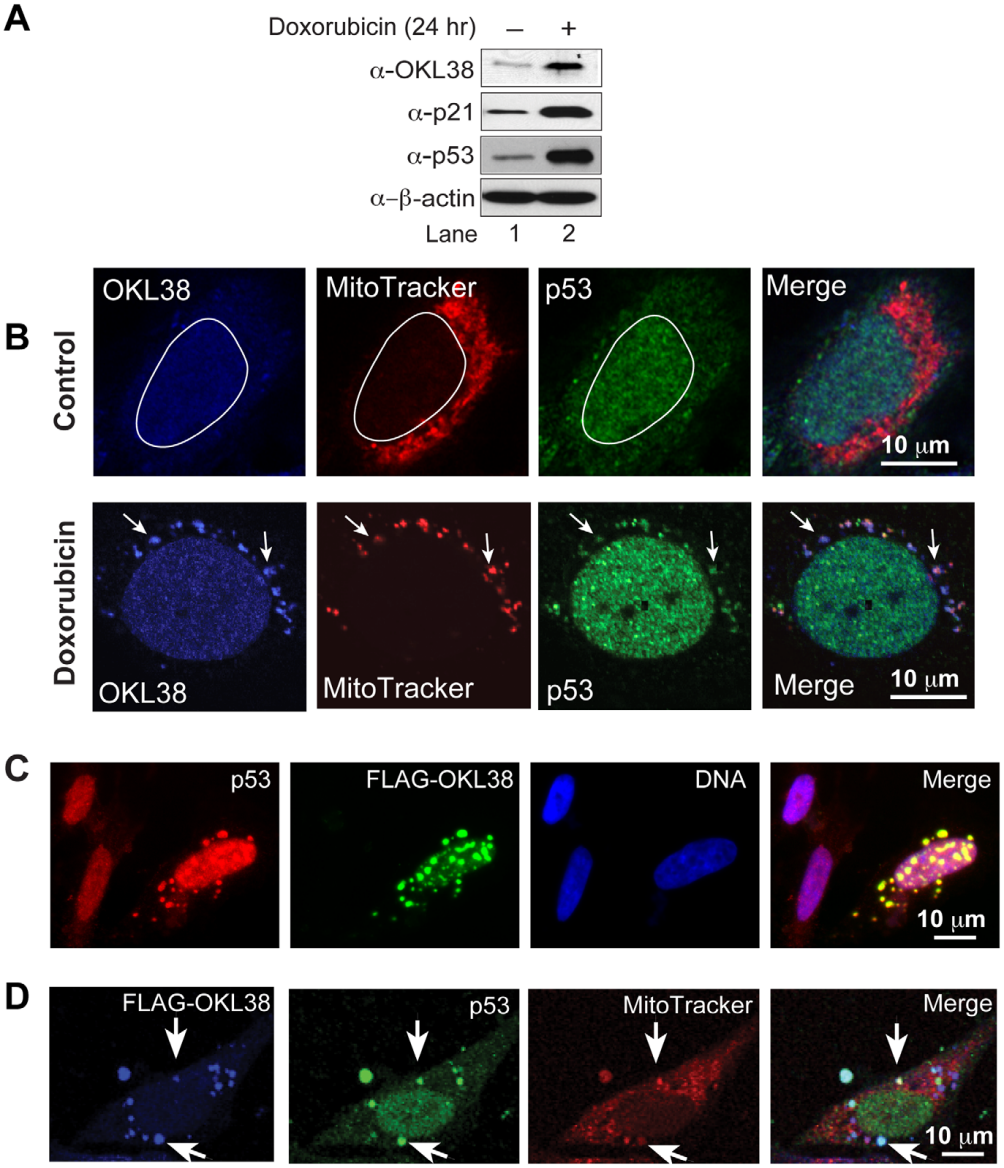
OKL38 and p53 colocalize in mitochondria. (A) Changes in OKL38, p21, and p53 protein levels after doxorubicin treatment were analyzed by Western blot. β-actin was probed as a loading control. (B) Upper panels: before DNA damage, confocal images showed that OKL38 (pseudocolored blue) and p53 (green) were enriched in the nucleus outlined with a irregular white circle in U2OS cells. Lower panels: after DNA damage by doxorubicin, both OKL38 (blue) and p53 (green) were detected in mitochondria stained by the MitoTracker Red dye. Arrows denote the mitochondria spots with OKL38 and p53 staining. (C) Fluorescent microscopy images showed that p53 (red) and OKL38 (green) colocalized in large speckles outside of nucleus (DNA staining in blue). (D) Confocal images showed that FLAG-OKL38 (blue) and p53 (green) colocalized with mitochondria (red) after FLAG-OKL38 overexpression in U2OS cells. Arrows denote the large mitochondria speckles with both FLAG-OKL38 and p53 localization.

We have previously found that OKL38 mRNA levels were induced by DNA damaging reagent treatment in U2OS cells [23]. To analyze whether OKL38 protein is induced by DNA damage, Western blot analyses were performed. An increase of OKL38 protein was detected in U2OS cells after treatment with doxorubicin for 24 hr (Figure 1A). Further, endogenous OKL38 was detected mainly in the nucleus in the U2OS cells before DNA damage by confocal imaging (Figure 1B, upper panels). In contrast, after doxorubicin treatment, OKL38 was detected in both nucleus and mitochondria speckles (Figure 1B, lower panels). Since p53 also plays a direct role in apoptosis in mitochondria [13]–[15], we next examined whether OKL38 and p53 colocalize in mitochondria. Immunostaining showed that p53 and OKL38 colocalize in a subset of mitochondria in U2OS cells after but not before DNA damage treatment (compare Figure 1B upper panels with lower panels).

FLAG-OKL38 expressed in U2OS cells was found to first accumulate in the nucleus and then enrich in mitochondria over time, and the localization of FLAG-OKL38 to mitochondria altered the morphology of mitochondria and induced apoptosis and cytochrome *c* release [23] (also see Figure S1). Next, we analyzed the effects of FLAG-OKL38 enrichment in mitochondria on the subcellular distribution of p53. When immunostaining was performed in U2OS cells after transfection with a FLAG-OKL38 expressing plasmid, FLAG-OKL38 was detected in many large cytoplasmic speckles that are also stained by the p53 antibody, suggesting that FLAG-OKL38 expression affects subcellular localization of p53 (Figure 1C). Furthermore, these large speckles stained by p53 and OKL38 antibodies are also stained by a mitochondria dye (MitoTracker red, Figure 1D, denoted by arrows) in triple labeling experiment, suggesting that OKL38 regulates p53-targeting to mitochondria.

### The p53^R72^ Polymorphic Allele More Efficiently Targets OKL38 to Mitochondria than the p53^P72^ Allele

**Figure 2:**
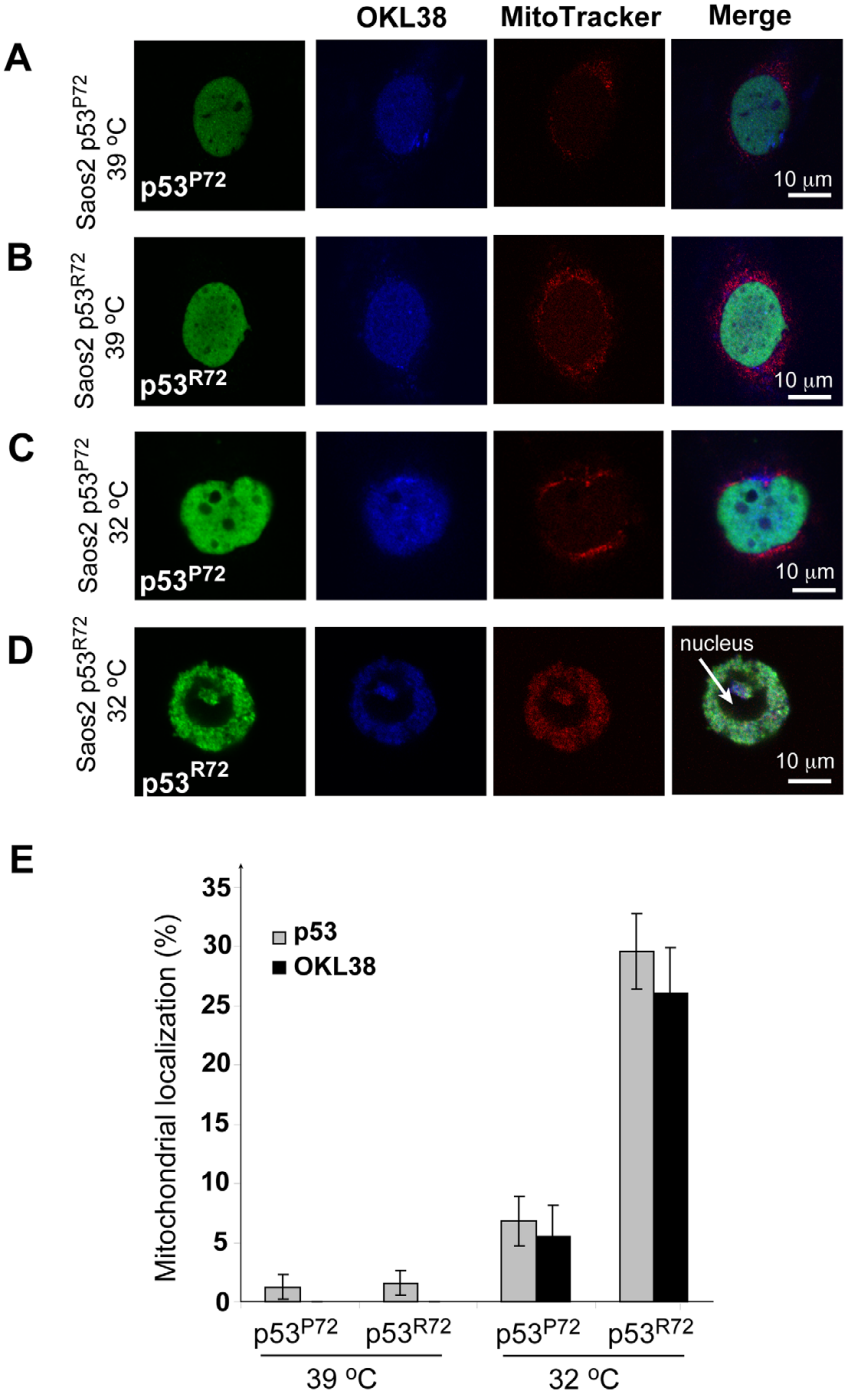
The p53^R72^ polymorphic allele has increased ability to target OKL38 to mitochondria than the p53^P72^ allele. (A) p53^P72^ (green) and OKL38 (pseudocolored blue) localized at the nucleus in Saos-2 cells at 39°C. (B) p53^R72^ (green) and OKL38 (blue) localized at the nucleus in Saos-2 cells at 39°C. (C) p53^P72^ (green) and OKL38 (blue) still localized at the nucleus in Saos-2 cells after temperature shift to 32°C. (D) p53^R72^ (green) and OKL38 (blue) colocalized with the staining of MitoTracker Red, a mitochondria dye, in Saos-2 cells after temperature shift to 32°C. (E) Percentages of cells with nuclear or mitochondrial localization of p53 and OKL38 were analyzed from three independent experiments. Standard deviations are shown (n = 3).

It has been reported that a common p53 polymorphic allele, p53^R72^, associates with the mitochondria and induces apoptosis markedly better than the p53^P72^ allele [22]. To test the effects of p53 mitochondria localization on OKL38, triple immunostaining of p53, OKL38, and mitochondria was performed in the p53^−/−^ Saos-2 cells stably transfected with plasmids expressing a temperature-sensitive p53 protein (Val 138-Ala) in *cis* with either Pro or Arg at amino-acid position 72 [22]. The p53 protein containing Val–138-Ala point mutation is inactive at 39°C, but has wild type conformation and activity after a temperature shift to 32°C [22]. At the 39°C nonpermissive temperature, OKL38 and p53 were mainly localized in the nucleus in Saos-2 cells expressing either p53^P72^ or p53^R72^ (Figure 2A and 2B). After temperature was shifted to 32°C for 24 hr, >93% of Saos-2 cells showed nuclear localization of p53^P72^ and OKL38 (Figure 2C), while mitochondrial localization of p53^P72^ and OKL38 was detected in ∼6.8% and ∼5% of cells, respectively (Figure 2E). In contrast, mitochondria localization of p53^R72^ and OKL38 were detected at higher rates of 29.6% and 26% respectively in Saos2 cells after temperature shift (Figure 2D and 2E), with the majority of these cells showing p53^R72^ and OKL38 colocalization in mitochondria (Figure 2D). Bar graphs in Figure 2E summarized changes in the percentages of cells showing mitochondria localization of p53 or OKL38 from three independent assays. Taken together, above results suggest that mitochondrial localization of p53 facilitates the recruitment of OKL38.

### OKL38 Interacts with p53 *in vivo* and *in vitro* via Multiple Domains

**Figure 3:**
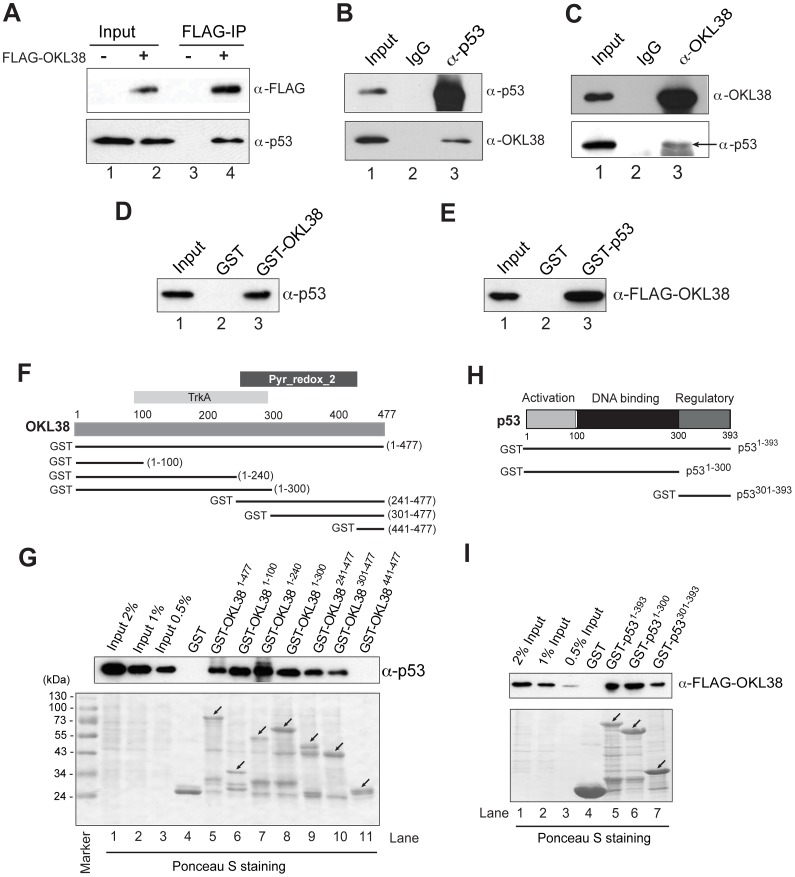
Interaction of OKL38 and p53 proteins. (A) p53 was co-immunoprecipitated by the M2 agarose beads together with the FLAG-OKL38 expressed in p53^+/+^ HCT116 cells, but not from the control cells without FLAG-OKL38 expression. (B) Nuclear extracts of U2OS cells were precipitated with normal mouse IgG or anti-p53 antibody and blotted with anti-p53 and anti-OKL38 antibodies. (C) Nuclear extracts of U2OS cells were precipitated with normal rabbit IgG or anti-OKL38 antibody and blotted with the anti-OKL38 and anti-p53 antibodies. Arrow denotes the position of p53. (D) GST-pull down experiments showed that GST-OKL38 beads retained p53 from nuclear extracts. (E) Similarly, GST-p53 beads retained FLAG-OKL38 expressed in Hela cells. (F) Illustration of OKL38 domain structures (TrkA and Pyr_Redox_2 domains) and the GST-truncation OKL38 constructs used to analyze OKL38 and p53 interaction. (G) Interaction of p53 with GST-OKL38 full length (residues 1–477) or its truncation derivatives in GST-pull down assays. Different percentages of input were loaded to serve as a control. (H) Illustration of p53 domain structures and its derivatives used in studying p53 and OKL38 interaction. (I) GST-pull down assays to detect interaction of FLAG-OKL38 with GST-p53 and its truncation derivatives.

The ability of OKL38 and p53 to facilitate each other in mitochondrial targeting prompted us to test whether these two proteins interact. We first performed immunoprecipitation experiments with the anti-FLAG M2 agarose beads using the p53^+/+^ HCT116 cell extracts with the FLAG-OKL38 expression. p53 was retained by the M2 agarose beads from the cells expressing FLAG-OKL38, but not from cells without FLAG-OKL38 expression (Figure 3A, lane 4 compared with lane 3). To test whether endogenous OKL38 interacts with p53, we performed co-immunoprecipitation experiments using U2OS cell extracts and found that OKL38 was co-immunoprecipitated by the p53 antibody but not by the normal mouse IgG (Figure 3B). Similarly, p53 was co-immunoprecipitated by the anti-OKL38 antibody but not by the normal rabbit IgG from the U2OS cell extracts (Figure 3C). Further, Glutathione-S-transferase (GST) -pull down experiments showed that GST-OKL38 but not GST beads retained p53 (Figure 3D). Similarly, GST-p53 but not GST beads interacted with FLAG-OKL38 (Figure 3E). Taken together, above results indicate that p53 and OKL38 interact.

OKL38 protein contains a potential TrkA domain (residues 100–240 with homology to flavoprotein involved in K^+^ transport) and a potential Pyr-Redox domain (residues 241–400 with homology to pyridine nucleotide-disulphide oxidoreductase) [27]. To test which part of OKL38 protein is important in mediating its interaction with p53, we generated GST-OKL38 and its N- and C-terminal truncation derivatives (illustrated in Figure 3F). GST-pull down experiments defined two minimal domains sufficient for interacting with p53 in OKL38, bearing residues 1–100 (Figure 3G lane 6) or residues 301–440 (Figure 3G, lanes 10 and 11). p53 protein has a N-terminal activation domain, a middle DNA binding domain, and a C-terminal regulatory domain [30] (Figure 3H). Furthermore, we found that GST-p53 and its derivative fusion proteins, including the N-terminal domain of p53 (residues 1–300) and its C-terminal regulatory domain (residues 301–393), pulled down FLAG-OKL38 expressed in HCT116 cells (Figure 3I). Taken together, above results indicate that OKL38 interacts with p53 via multiple domains.

Since p53^R72^ targets OKL38 to mitochondria more efficiently than p53^P72^, we analyzed if the N-terminal domain of p53 with Pro72 or Arg72 differs in their OKL38 binding ability. We found that GST-p53^1–99-R72^ and GST-p53^1–99-P72^ did not show detectable FLAG-OKL38 binding (Figure S2). It has been found that the p53^R72^ allele interacts with a mitochondria integral protein Bak stronger than the p53^P72^ allele [14]. As such, the higher mitochondria localization frequency of p53^ R72^ than p53^ P72^ might contribute to its ability to target OKL38 to mitochondria.

### Multiple Domains of OKL38 Mediate its Subcellular Localization and its Regulation of Mitochondrial Morphology

**Figure 4:**
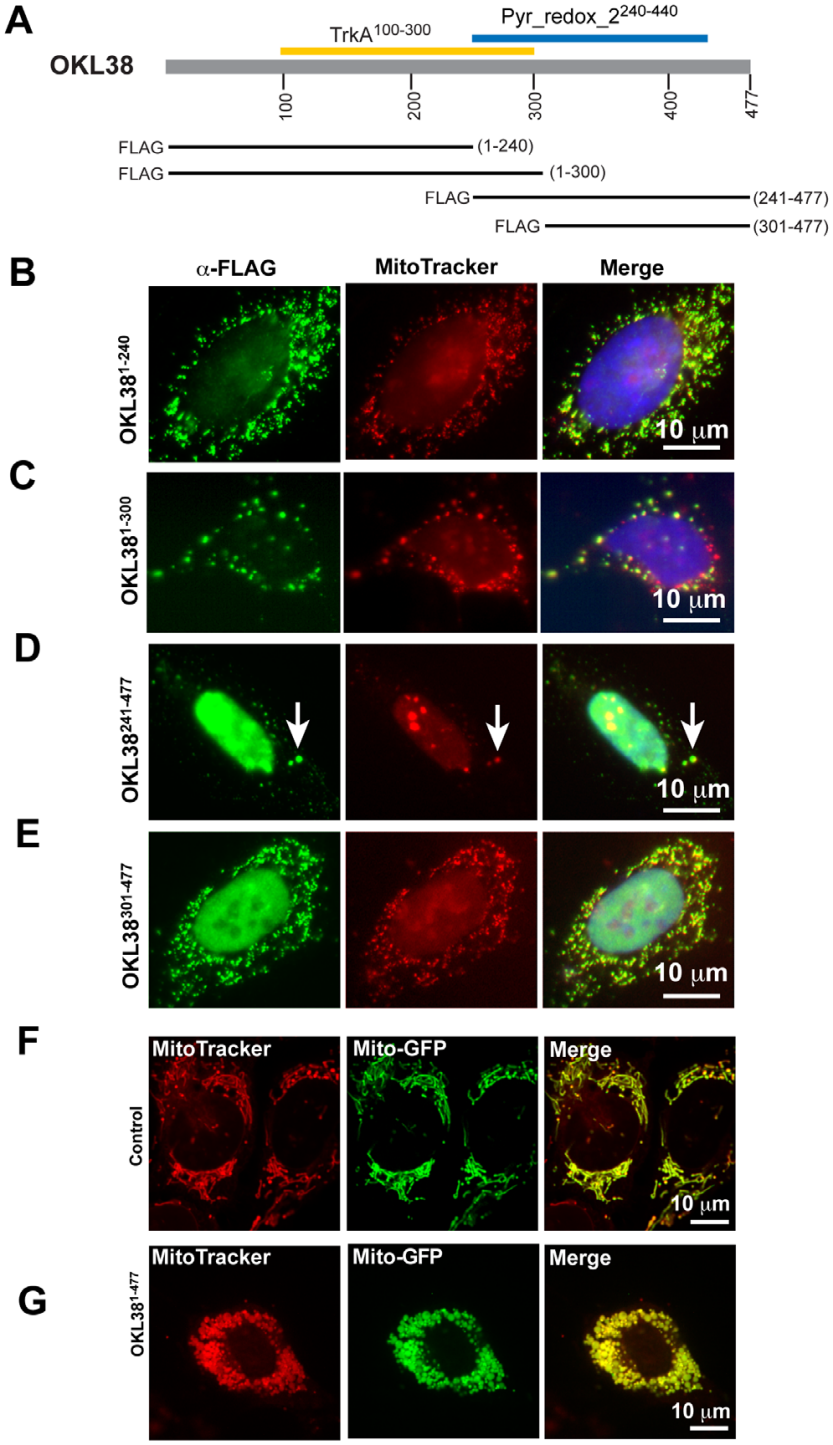
Subcellular localization of OKL38 truncation derivatives. (**A**) Schematic drawing of the OKL38 domain structure and FLAG-OKL38 truncations used to analyze subcellular distribution. (B–C) Preferential mitochondrial localization of FLAG-OKL38^1–240^ (B) and FLAG-OKL38^1–300^ (C). (D–E) Nuclear and mitochondrial localization of FLAG-OKL38^241–477^ (D, arrows denote mitochondria) and FLAG-OKL38^301–477^ (E). Also see figure S4 for more examples of subcellular localization of FLAG-OKL38 truncations. (F–G) The morphology of mitochondria in live cells was detected using a GFP-mito reporter protein in control U2OS cells (F) or in cells co-transfected with the full length FLAG-OKL38 construct (G). Also see figure S5 for more examples of mitochondria changes in cells co-transfected with FLAG-OKL38 and GFP-mito.

To analyze domains of OKL38 important for its subcellular localization and function, we expressed FLAG-OKL38 and its truncation derivatives in U2OS cells. Consistent with previous results [23], FLAG-OKL38 showed either mainly nuclear localization (Figure S1A) or mainly mitochondrial localization (Figure S1B) in different subset of cells. Moreover, these large structures induced by FLAG-OKL38 expression are positively stained by an antibody against the mitochondrial marker protein cytochrome *c* oxidase (COX) I (Figure S3), suggesting these structures are derived from mitochondria. In contrast to full length OKL38, the FLAG-OKL38^1–240^ and FLAG-OKL38^1–300^ truncations preferentially localize to mitochondrial compartment (Figure 4B, 4C, and Figure S4). Moreover, targeting the N-terminal portions of OKL38 to mitochondria induced the formation of large mitochondria speckles (Figure S4). On the other hand, the FLAG-OKL38^241–477^ truncation preferentially localizes to the nucleus (Figure 4D, Figure S4) while FLAG-OKL38^301–477^ truncation has a decreased frequency for nuclear localization compared with the FLAG-OKL38^241–477^ construct (Figure 4E, Figure S4). Furthermore, both FLAG-OKL38^241–477^ and FLAG-OKL38^301–477^ truncations affect mitochondrial morphology after their mitochondrial localization (Figure S4C and S4D). The percentage of cells expressing OKL38 or its truncations with various degrees of localization to nucleus, mitochondrion, or both was numerated (Figure S4E, over 250 transfected cells was scored for subcellular localization by two independent observers). Taken together, above results suggest that multiple domains of OKL38 regulate its subcellular localization and function in controlling mitochondrial morphology, likely via their interaction with other mitochondrial proteins.

To further analyze OKL38-mediated mitochondria morphology change, we performed live cell imaging after co-transfection of the FLAG-OKL38 construct with a GFP-mito reporter construct. Spinning disk confocal live imaging showed an elongated fiber-like mitochondria structure in U2OS cells transfected with the GFP-mito reporter alone (Figure 4F). In contrast, upon co-transfection with the FLAG-OKL38 construct, mitochondria fragmentation and formation of large speckles were detected (Figure 4G and Figure S5A–B). These results further support that OKL38 plays a role in the regulation of mitochondria structure.

### OKL38 Induces Megamitochondria Formation and Accumulation of Reactive Oxygen Species (ROS)

**Figure 5:**
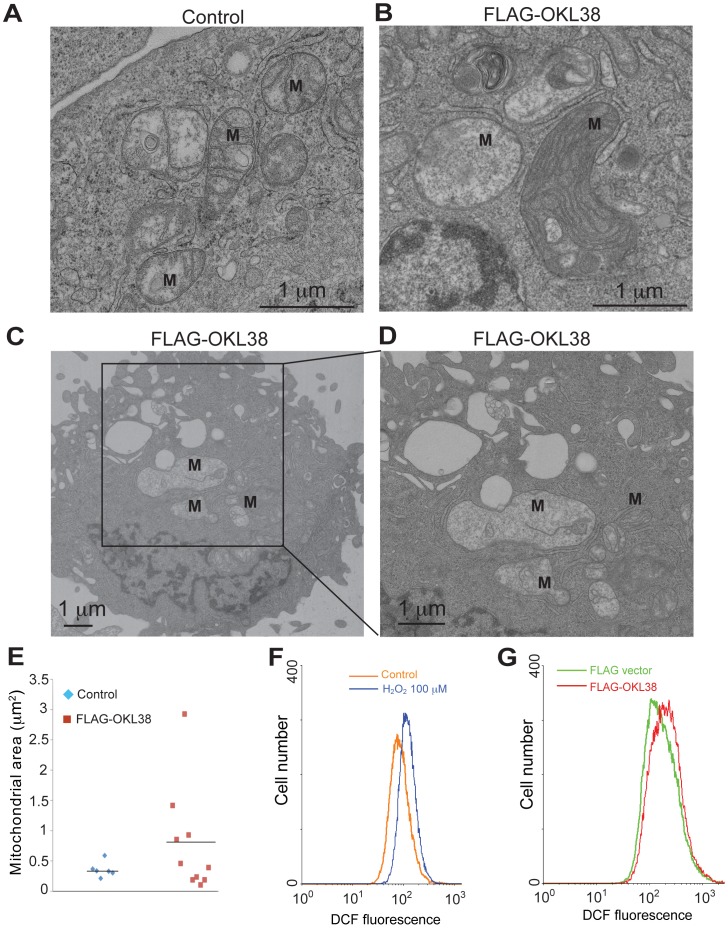
OKL38 expression induced formation of megamitochondria and ROS production. (A) Ultrastructure analyses of mitochondria (M) using TEM in U2OS cells. (B–D) Ultrastructure analyses of mitochondria (M) in U2OS cells after OKL38 expression. Note the enlargement of mitochondria and the alteration of cristae structures. (E) The surface mitochondria areas in TEM images were analyzed by NIH image J program. The occurrence of megamitochondria was only observed in U2OS cells after OKL38 expression. (F) Flow cytometry analyses of cellular ROS levels in U2OS cells after H_2_O_2_ treatment. (G) Flow cytometry analyses of cellular ROS levels after FLAG-OKL38 expression. The DCF fluorescence signals were increased after H_2_O_2_ treatment or FLAG-OKL38 expression.

The morphology of the mitochondria is regulated by the homeostasis of fission and fusion cycles [19]. The formation of big mitochondria (also called megamitochondria) plays a role in cell’s ability to cope with reactive oxygen species and cell death with important pathological and physiological implications [31]. To further analyze mitochondria ultrastructure changes, we performed transmission electron microscopy (TEM) analyses of U2OS cells without or with forced OKL38 expression. Without OKL38 expression, mitochondria are detected as double membrane structures with cristae formed by the inner mitochondria membrane (Figure 5A, ‘M’ denotes mitochondria). In contrast, after OKL38 expression, enlarged mitochondria were observed (Figures 5B–D), which also showed changes in the shape and number of cristae structures. The mitochondrial surface area in control and OKL38 expressing cells were measured using the NIH image J program. We found the median size of the mitochondrial areas was significantly increased (Figure 5E), indicating that OKL38 expression leads to the formation of megamitochondria.

**Figure 6:**
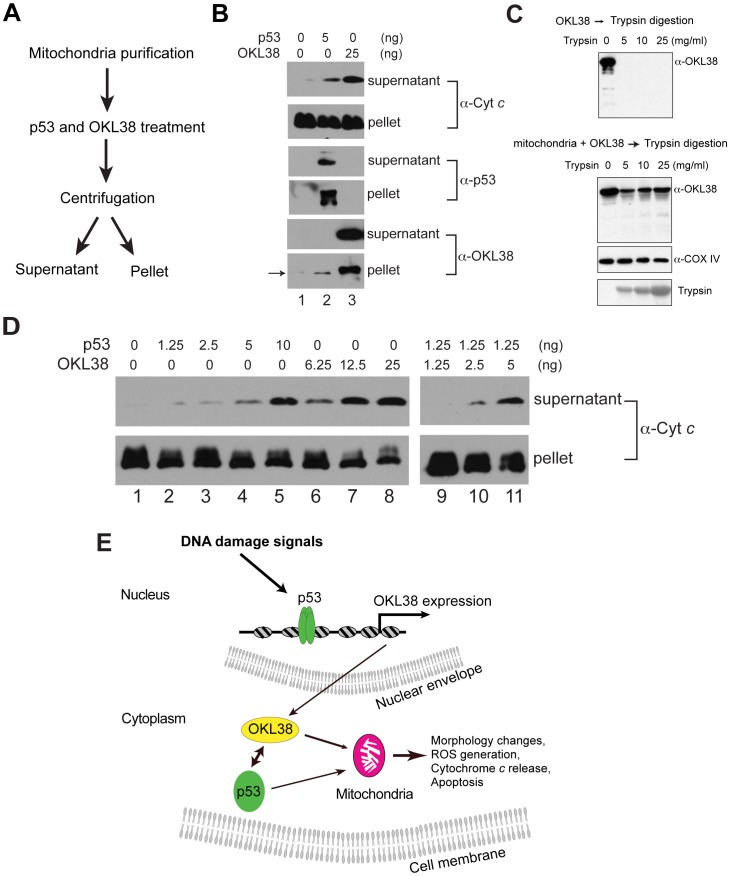
OKL38 and p53 induced cytochrome *c* release from purified mitochondria *in vitro*. (A) Biochemical scheme used to analyze the effect of p53 and OKL38 on cytochrome *c* release from mitochondria. (B) Effects of p53 and OKL38 on cytochrome *c* release (top 2 panels), and the detection of p53 (middle 2 panels) and OKL38 (bottom 2 panels) in both soluble and pellet fractions by Western blot. Arrow denotes low levels of endogenous OKL38 in mouse liver mitochondria. (C) Western blot analyses of OKL38 digestion by trypsin without (upper panel) or with prior incubation with mitochondria (Lower panel). COX IV protein was monitored to show that mitochondrial protein is protected from trypsin digestion. The amount of trypsin in the reaction was detected by Ponceau S staining. (D) Western blot analyses of the amount of cytochrome *c* in the supernatant and pellet fractions after mitochondria were incubated with p53 and/or OKL38 at various concentrations. (E) A model of the function of OKL38 and its cooperation with p53 during DNA damage to regulate mitochondria-mediated cell death. Our data favor a model that after DNA damage, p53 activates the expression of OKL38. On the other hand, OKL38 protein can interact with p53 in cytoplasm and target each other to mitochondria to regulate mitochondrial morphology, function, and cell death.

Reactive oxygen species (ROS) plays an important role in mitochondria function and apoptosis. To test whether OKL38 affects mitochondria function and ROS production, we measured the cellular ROS levels using flow cytometry. As a positive control for ROS measurement, we used H_2_O_2_ to treat U2OS cells. H_2_O_2_ treatment increased cellular levels of ROS, showing a peak shift compared with untreated cells (Figure 5F). After transient transfection to express FLAG-OKL38 in U2OS cells, we found that cellular ROS levels were increased compared with cells transfected with the pIRES vector alone (Figure 5G), indicating that OKL38 elevates cellular ROS generation.

### OKL38 and p53 Induce Cytochrome *c* Release from Purified Mitochondria *in vitro*

Upon incubation with mitochondria, p53 was found to facilitate cytochrome *c* release [13], [15]. Although we have previously found that OKL38 overexpression in U2OS cells can ultimately induce cytochrome *c* release using immunostaining assays [23], whether OKL38 plays a direct role in mediating cytochrome *c* release is unclear. Using a biochemical assay strategy (Figure 6A), we found that affinity purified p53 or OKL38 protein can induce cytochrome *c* release from purified mouse liver mitochondria (Figure 6B). By quantification of the amount of cytochrome *c* in the pellet and in the supernatant, we found that the increase of cytochrome *c* is concomitant with an obvious decrease of cytochrome *c* in the pellet (Figure S6A). Additionally, both p53 and OKL38 can partition to the insoluble pellet fraction upon incubation with mitochondria (Figure 6B). To analyze whether OKL38 loosely associates with mitochondria outer membrane or tightly associates with mitochondria compartments, we performed trypsin digestion experiments. Affinity purified OKL38 can be fully digested by trypsin as shown by Western blot (Figure 6C, upper panel). In contrast, after incubation with mitochondria, a fraction of OKL38 is resistant to trypsin digestion (Figure 6C, lower panels), suggesting that OKL38 may translocate into mitochondria and/or tightly associated with mitochondria membrane components thereby being inaccessible to trypsin digestion. Furthermore, we found that p53 (Figure 6D, lanes 2–5) and OKL38 (Figure 6D, lanes 6–8) induced cytochrome *c* release in a dose dependent manner. When a combination of p53 and OKL38 was incubated with mitochondria, we found that lower concentrations of p53 and OKL38 together (Figure 6C, lanes 10–11) induced more efficient cytochrome *c* release than single protein used at the same concentration, suggesting p53 and OKL38 might cooperate to induce cytochrome *c* release.

Although many groups use the mannitol and sucrose containing cytochrome *c* release buffer [13], [32], these compounds increase the nonionic osmolarity of the buffer. As such, we further tested the ability of OKL38 to induce cytochrome *c* release using a near isotonic KCl-based cytochrome *c* release buffer similar as previously described by other groups [15], [33]. We found that OKL38 induced mitochondrial cytochrome *c* release in the KCl-based buffer in a dose dependent manner (Figure S6B), further supporting a role of OKL38 in regulating mitochondrial function by impacting on cytochrome *c* release.

## Discussion

We have shown that *OKL38* is a novel downstream target gene of the tumor suppressor p53, which plays a proapoptotic role in mitochondria [23]. Here, we found that OKL38 and p53 facilitate each other in mitochondrial targeting by protein-protein interaction. Our further analyses of OKL38 functioning domains found that multiple regions of OKL38 mediate its interaction with p53, its localization to mitochondria, and its ability in modulating mitochondrial morphology. Our data support a model that during cellular response to DNA damage, OKL38 associates with mitochondria and facilitates p53 to regulate apoptosis by regulating the homeostasis of ROS and cytochrome *c* release (Figure 6E).

In cells without DNA damage treatment, p53 is a short lived protein, and its stability is regulated by ubiquitination and subsequent protein degradation [34]. After DNA damage, p53 is phosphorylated at particular Ser residues (e.g., Ser15), which weakens its interaction with the E3 ligase (e.g., MDM2) thereby stabilizing p53 and allowing p53 to turn on numerous genes in nucleus [35].

Besides its well-characterized function as a transcription factor, p53 also plays a direct role in apoptosis induction in mitochondria [13]–[15]. p53 activates the proapoptotic proteins, such as Bax [13] and Bak [14], to change the permeability of the mitochondrial outer membrane thereby inducing cytochrome *c* release. Although the subcellular localization of p53 will have profound effects on its function, it is currently unclear how the localization of p53 to nucleus or mitochondria is regulated. Intriguingly, a p53 polymorphic allele, p53^R72^, associates with mitochondria and induces cell apoptosis more efficiently compared with the p53^P72^ allele [22]. Additionally, the p53^P72^ allele occurs more frequently in human population living near the equator [36], [37], suggesting this p53^P72^ allele is evolutionarily and environmentally favored in certain population.

We report here that OKL38 and p53 facilitate each other to localize to mitochondria. First, forced expression of FLAG-OKL38 induced the change of mitochondria morphology and also the localization of p53 to the same mitochondria speckles. Conversely, the presence of the functional p53^R72^ protein induced a translocation of OKL38 from nucleus to mitochondria. The interaction of OKL38 and p53 suggests that these two proteins may regulate each other in mitochondria localization and apoptosis induction. The mechanisms underlying OKL38 interaction with p53 in regulating apoptosis and the function of OKL38 in inducing cytochrome *c* release require further investigation. A possible scenario is that OKL38 interacts with other proteins in addition to p53 in mitochondria to control important mitochondrial functions.

The loss of OKL38 expression has been correlated with tumorigenesis in kidney and liver, suggesting OKL38 plays a role during tumorigenesis [28], [29]. The notion that OKL38 is suppressive for tumor development is in agreement with several observations that OKL38 is inhibitory for cell growth while promotes cell death [23], [27], [28]. Protein sequence alignment has identified a couple of putative functioning domains of OKL38, notably the N-terminal TrkA domain (residues 100–240) with homology to flavoproteins involved in K^+^ transport, and the C-terminal Pyr_Redox domain homologous to pyridine nucleotide-disulphide oxidoreductases [27]. Albeit with different efficacy, our studies have found that both the N- and C-terminal truncations of OKL38 can be targeted to mitochondria to regulate mitochondria morphology. Sequence analyses do not find a canonical mitochondrial targeting sequence in OKL38. Given that multiple truncation derivatives of OKL38 can be targeted to mitochondria, our data favor a view that OKL38 is likely targeted to mitochondria via interaction with other mitochondrial proteins. Currently, the biochemical function of OKL38 remains unclear. It is possible that both N- and C-terminal domains of OKL38 interact with p53 or other mitochondrial proteins to remodel the mitochondrial morphology, regulate ROS production, and facilitate cytochrome *c* release during stress. These fascinating aspects of OKL38 in mitochondria morphology and function regulation deserve future studies.

In summary, we propose a model that p53 and OKL38 function together to keep the homeostasis of cell growth and death and to cope with cellular damaging signals by tipping the balance toward apoptosis under stress and DNA damage conditions. A decrease of OKL38 in tumor cells may offer growth advantage by allowing accelerated cell growth and decreased cell death.

## Materials and Methods

### Plasmids

The pIRES-FLAG-OKL38 construct was previously described [23]. OKL38 cDNA and its truncation derivatives were cloned in the pGEX4T1 or the pIRES vector for expression in *E.coli* strain BL21 and mammalian cells, respectively. Constructs were confirmed by DNA sequencing at the Nucleic Acid Facility at the Pennsylvania State University.

### Cell Culture and Treatments

Osteosarcoma U2OS cells were cultured in DMEM medium supplemented with 10% FBS and 1% Penicillin-Streptomycin in a 5% CO_2_ incubator at 37°C. Doxorubicin was used at 0.4 µM to induce DNA damage in U2OS cells. Osteosarcoma Saos-2 cells with stable p53^R72^ or p53^P72^ expression were maintained in DMEM medium supplemented with 10% FBS, 1% Penicillin-Streptomycin, and 0.4 mg/ml G418 at 39°C or 32°C as specified.

### Nuclear Extract Preparations, GST-pull Down, Co-immunoprecipitation

Nuclear extracts were prepared essentially as previously described [12]. GST-pull down was performed following a previously described protocol [38]. For co-immunoprecipitation with FLAG-OKL38, M2 agarose beads (Sigma, A2220) were incubated with the nuclear extracts at 4°C for overnight, and washed three times with the medium salt buffer (20 mM Tris-HCl, pH 7.3, 300 mM KCl, 1.5 mM MgCl_2_, 0.2 mM EDTA, freshly supplemented with protease inhibitors) and finally washed with TBS. Co-immunoprecipitation experiments with p53 and OKL38 antibodies were performed essentially as previously described [38].

### Live Imaging, Immunostaining, Confocal and Electron Microscopy

Saos-2-p53 cells with R72 variant or P72 variant were grown on coverslips at 39°C for 24 hr, then shifted the temperature to 32°C for another 24 hr. For mitochondria staining, U2OS or Saos-2 cells were incubated with 150 nM of MitoTracker red (Invitrogen, M7512) for 45 min, and then fixed in 4% paraformaldehyde in PBS and permeabilized in 0.1% Triton X-100. Fixed cells were blocked with 2% BSA and incubated with primary antibodies: anti-p53 (Sigma, BP53-12, 1∶500), affinity purified anti-OKL38 (1∶100), or anti-COX I (Santa Cruz, sc-58347, 1∶10), for overnight at 4°C. Cells were then reacted with secondary antibodies, Alexa 488-conjugated goat anti-mouse IgG (1∶800) or Alexa 633-conjugated goat anti-rabbit IgG (1∶800), for 2 hr at room temperature. Images were captured by an inverted FV1000 confocal microscope at the Flow Cytometry facility at the Pennsylvania State University, and further processed in the Adobe Photoshop and Illustrator programs. For transmission electron microscopy analyses of mitochondria morphology, cells were fixed with glutaraldehyde, processed, and analyzed at the Pennsylvania State Electron Microscopy Facility using a Jeol JEM 1200 EX II electron microscope operated at 80 kV.

The GFP-mito reporter plasmid was obtained from Addgene (# 23348). The reporter plasmid was transfected alone or together with the FLAG-OKL38 construct into U2OS cells using the Lipofectamine2000 (Invitrogen) according to the manufacturer’s instructions. After transfection, U2OS cells are cultured for another 24 hr to allow protein expression before confocal microscopy imaging. Imaging was performed essentially as previously described [39]. Briefly, imaging was done on a Yokogawa CSUX1spinning disk system completed with a Photometrics QuantEM:512SC CCD camera, DMI6000 Leica motorized microscope, and a Leica 100×/1.4 n.a. oil objective. An ATOF laser with 491/561 nm laser line enabled fast shuttering and switching between different excitations. Band-pass filters (520/50 nm for GFP; 620/60 nm for MitoTracker) were used for emission filtering. All image acquisition settings were kept the same during the image collection.

### Measurement of Reactive Oxygen Species (ROS) by Flow Cytometry

U2OS Cells transfected with pIRES-FLAG vector or pIRES-FLAG-OKL38 plasmid for 24 hr were harvested by trypsin with centrifugation at 1000 rpm for 5 min. The cell pellet was resuspended in 1 ml of pre-warmed DMEM and treated with 10 µM of 2,7- dichlorofluorescein (DCF, Sigma-Aldrich, D6665) for 30 min in the dark at 37°C. The fluorescence was assessed by FC500 flow cytometer (Beckmann-Coulter) at the Flow Cytometry Facility of the Pennsylvania State University and analyzed with WinMDI 2.9 software. As negative and positive controls for ROS detection, the untransfected cells were incubated with DCF and then with or without 100 µM of H_2_O_2_ for 15 min in the dark at room temperature.

### Mitochondria Purification and Analysis of Cytochrome *c* Release

Mitochondria were isolated from mouse livers largely as previously described [13]. Briefly, mouse liver was dissected and washed with PBS three times. Washed tissue is dounce-homogenized in mitochondria isolation buffer (10 mM HEPES-KOH pH 7.4, 200 mM mannitol, 250 mM sucrose, 1 mM EDTA, 1 mM EGTA, 0.1% BSA, and protease inhibitors) with a tight pestle for 72 times. Large cell debris was removed by centrifugation at 700 g for 10 min at 4°C. Mitochondria in the supernatant were collected by centrifugation at 7, 000 g for 10 min at 4°C. Mitochondria were washed once with the cytochrome *c* release buffer (10 mM HEPES-KOH pH 7.4, 200 mM mannitol, 68 mM sucrose, 100 mM KCl, 1 mM EDTA, 1 mM EGTA, 5 mM succinate, 2 mM ATP, and protease inhibitors). FLAG-p53 and His6-OKL38 fusion proteins were expressed in and purified from *E.coli*. FLAG-p53 was purified by M2-agarose beads (Sigma, A2220). His6-OKL38 was purified using Ni-NTA beads (QIAGEN, 1018244) and further purified and desalted using Superose 6 column 10/300 GL (GE healthcare, 17-5172-01). Purified proteins were incubated with mitochondria at concentrations indicated in cytochrome *c* release buffer for 60 min at 37°C. Samples were centrifuged at 7, 000 g for 10 min to collect the supernatants. Pellets were further washed twice in cytochrome *c* release buffer. Proteins in the supernatants and pellets were analyzed by SDS-PAGE and Western blot with anti-cytochrome *c* (BD Pharmingen, 556433, 1∶1000), anti-p53 (Sigma, BP53–12, 1∶2000), and affinity purified anti-OKL38 (1∶500) antibodies. Western blot signals were detected using the Lumi-Light^Plus^ Western blotting substrate (Roche Inc.). To quantify the amount of cytochrome *c*, the NIH image J program was used. To measure cytochrome *c* release in a more isotonic buffer, purified mitochondria were washed with the KCl cytochrome *c* release buffer (10 mM Hepes-KOH pH 7.4, 125 mM KCl, 5 mM succinate, 5 mM KH_2_PO_4_, 1 mM EDTA, 1 mM EGTA, 2 mM ATP, freshly supplemented with proteinase inhibitors). The OKL38 protein was incubated with mitochondria at concentrations indicated in the KCl cytochrome *c* release buffer for 60 min at room temperature. Samples were centrifuged at 7, 000 g for 10 min to collect the supernatants. Pellets were further washed in the KCl cytochrome *c* release buffer. Proteins in the supernatants and pellets were analyzed by Western blot.

### *In vitro* Partial Trypsin Digestion Assay

Mouse liver mitochondria were isolated freshly as cytochrome *c* release assay and incubated for 1 hr at 37°C in the presence of purified His6-OKL38 protein. After centrifuged at 7000 g for 10 min at 4°C, the mitochondria pellets were washed once and incubated with trypsin at concentrations indicated for 15 min at 37°C. His6-OKL38 without mitochondria incubation was digested under similar condition as a control. All reactions were stopped by adding 6× SDS-PAGE sample buffer and analyzed by western blot using affinity purified anti-OKL38 (1∶500) and anti-COX IV (Abcam, ab16056, 1∶2000) antibodies.

## Supporting Information

Figure S1 **Subcellular distribution of FLAG-OKL38 in U2OS cells.** (A) In a subset of transfected cells, FLAG-OKL38 showed nuclear staining. (B) In another subset of cells, FLAG-OKL38 staining overlapped with the mitochondrial dye, MitoTracker staining.
(TIF)

Figure S2 **Interaction of p53 N-terminus with OKL38.** The binding of full length GST-p53 fusion protein to FLAG-OKL38 (lane 5) was detected but not GST-p53 residues 1–99 truncation derivatives containing either a P72 (lane 6) or a R72 (lane 7) residue.
(TIF)

Figure S3 **FLAG-OKL38 staining overlaps with mitochondrial protein COX I.** (A) Large cytoplasmic speckles enriched with FLAG-OKL38 were stained by anti-OKL38 rabbit pAb (green colored). These large speckles are also positively labeled with the COX I mouse mAb antibody (red colored). The overlap between FLAG-OKL38 and COX I staining indicate that these large speckles are formed by mitochondria. (B) COX I staining overlaps with that of the MitoTracker staining in the large speckles formed after FLAG-OKL38 transfection. (C) Large cytoplasmic speckles induced by FLAG-OKL38^241–477^ were stained by anti-OKL38 rabbit pAb (green colored) as well as the COX I mouse mAb antibody (red colored).
(TIF)

Figure S4 **Subcellular distribution of FLAG-OKL38 truncations in U2OS cells.** (A) OKL38 residues 1–240. (B) OKL38 residues 1–300. (C) OKL38 residues 241–477. (D) OKL38 residues 301–477. (E) Over 250 cells from independent experiments were scored by two observers for subcellular localization of OKL38 and its truncation derivatives. Percentages of cells with mainly mitochondrial, mainly nuclear, or both mitochondrial and nuclear OKL38 localization are shown in the bar graphs.
(TIF)

Figure S5 **Effects of FLAG-OKL38 on the GFP-mito reporter distribution.** (A–B) FLAG-OKL38 full length construct was co-transfected with the GFP-mito reporter construct in U2OS cells. Fragmentation of mitochondria and formation of large mitochondria speckles were detected in live cell imaging analyses.
(TIF)

Figure S6 **Relative abundance of cytochrome** ***c*** **in pellet and supernatant after p53 or OKL38 incubation.** (A) The amount of cytochrome *c* in the supernatant (upper panel) or in the pellet (lower panel) was detected by Western blot. The relative signal under each treatment condition was measured using the NIH Image J program. With the increased amount of cytochrome *c* released, a concomitant decrease of cytochrome *c* from the pellet was detected. (B) The effect of OKL38 on mitochondrial cytochrome *c* release was tested using an isotonic buffer containing 125 mM KCl and other salts. The amount of cytochrome *c* in the supernatant or in the pellet (two upper panels) was detected by Western blot. The relative signal was measured using the NIH Image J program. With the increased amount of OKL38, a concomitant increase of cytochrome *c* release was detected. The amount of OKL38 in the supernatant or the pellet (two lower panels) was also monitored by Western blot. Arrow denotes the recombinant OKL38 detected in the mitochondrial pellet.
(TIF)
