# Supplementary figures and images for: Interaction of OKL38 and p53 in Regulating Mitochondrial Structure and Function
Source: PLoS One. 2012 Aug 17;7(8):e43362. doi: 10.1371/journal.pone.0043362 (PMC3422280; doi:10.1371/journal.pone.0043362)

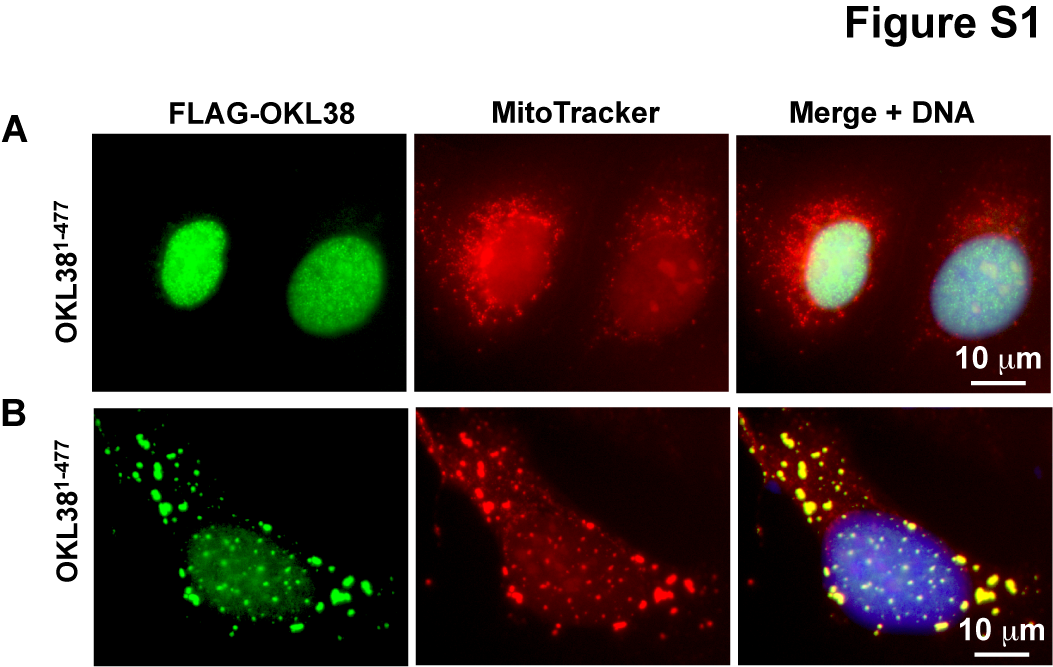

Supplement: Figure S1 — Subcellular distribution of FLAG-OKL38 in U2OS cells. (A) In a subset of transfected cells, FLAG-OKL38 showed nuclear staining. (B) In another subset of cells, FLAG-OKL38 staining overlapped with the mitochondrial dye, MitoTracker staining. (TIF) [file pone.0043362.s001.tif]

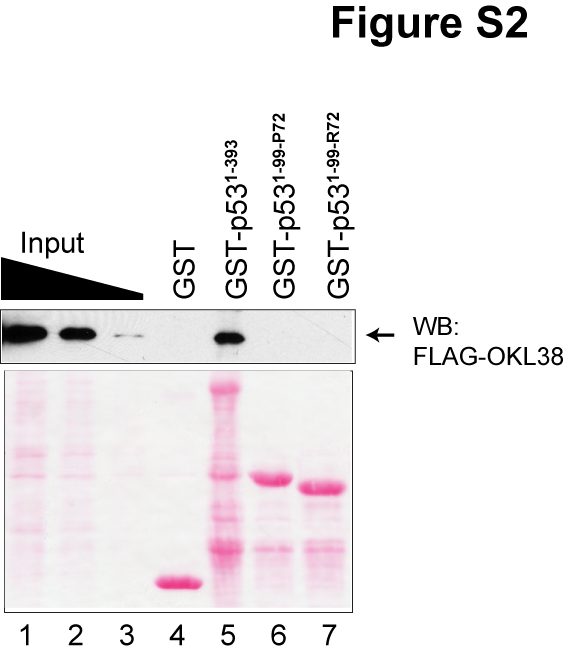

Supplement: Figure S2 — Interaction of p53 N-terminus with OKL38. The binding of full length GST-p53 fusion protein to FLAG-OKL38 (lane 5) was detected but not GST-p53 residues 1–99 truncation derivatives containing either a P72 (lane 6) or a R72 (lane 7) residue. (TIF) [file pone.0043362.s002.tif]

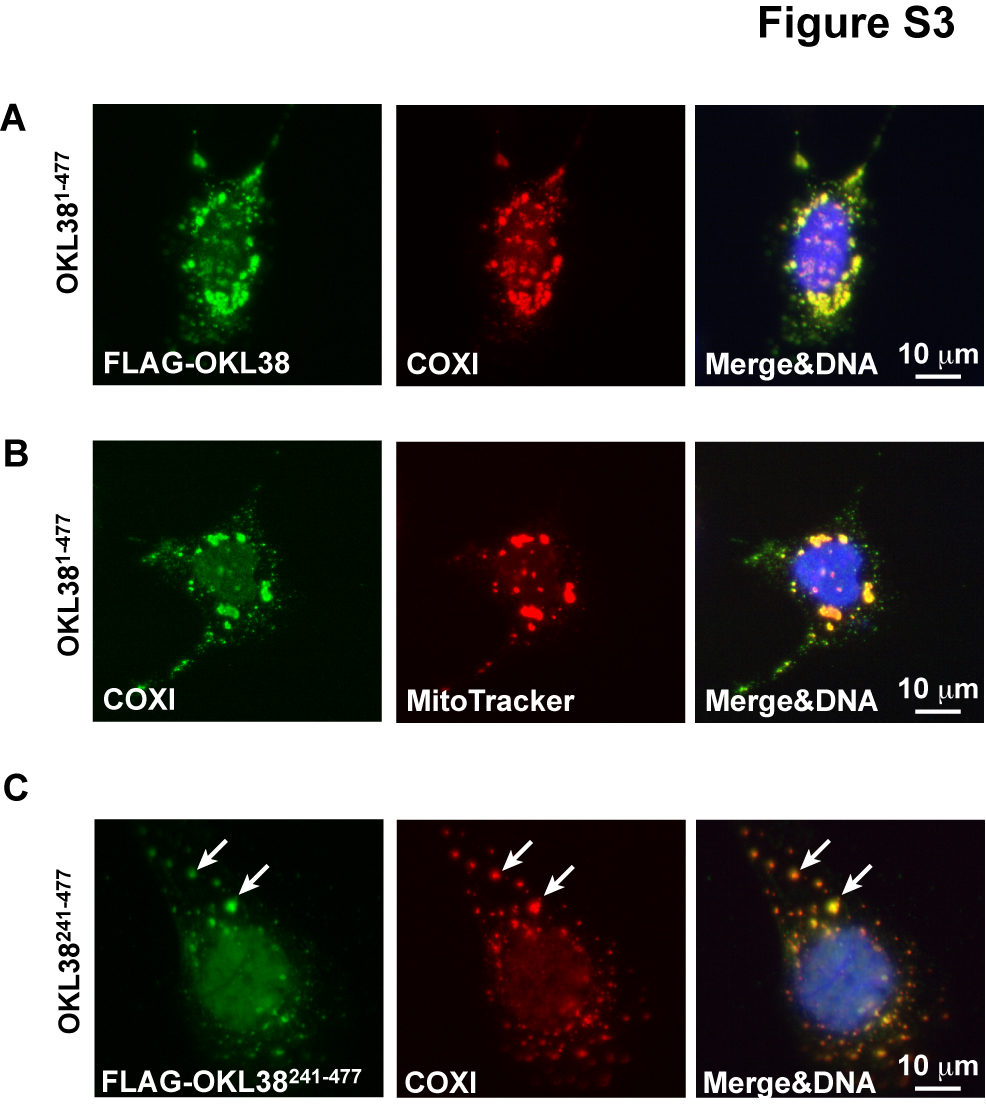

Supplement: Figure S3 — FLAG-OKL38 staining overlaps with mitochondrial protein COX I. (A) Large cytoplasmic speckles enriched with FLAG-OKL38 were stained by anti-OKL38 rabbit pAb (green colored). These large speckles are also positively labeled with the COX I mouse mAb antibody (red colored). The overlap between FLAG-OKL38 and COX I staining indicate that these large speckles are formed by mitochondria. (B) COX I staining overlaps with that of the MitoTracker staining in the large speckles formed after FLAG-OKL38 transfection. (C) Large cytoplasmic speckles induced by FLAG-OKL38241–477 were stained by anti-OKL38 rabbit pAb (green colored) as well as the COX I mouse mAb antibody (red colored). (TIF) [file pone.0043362.s003.tif]

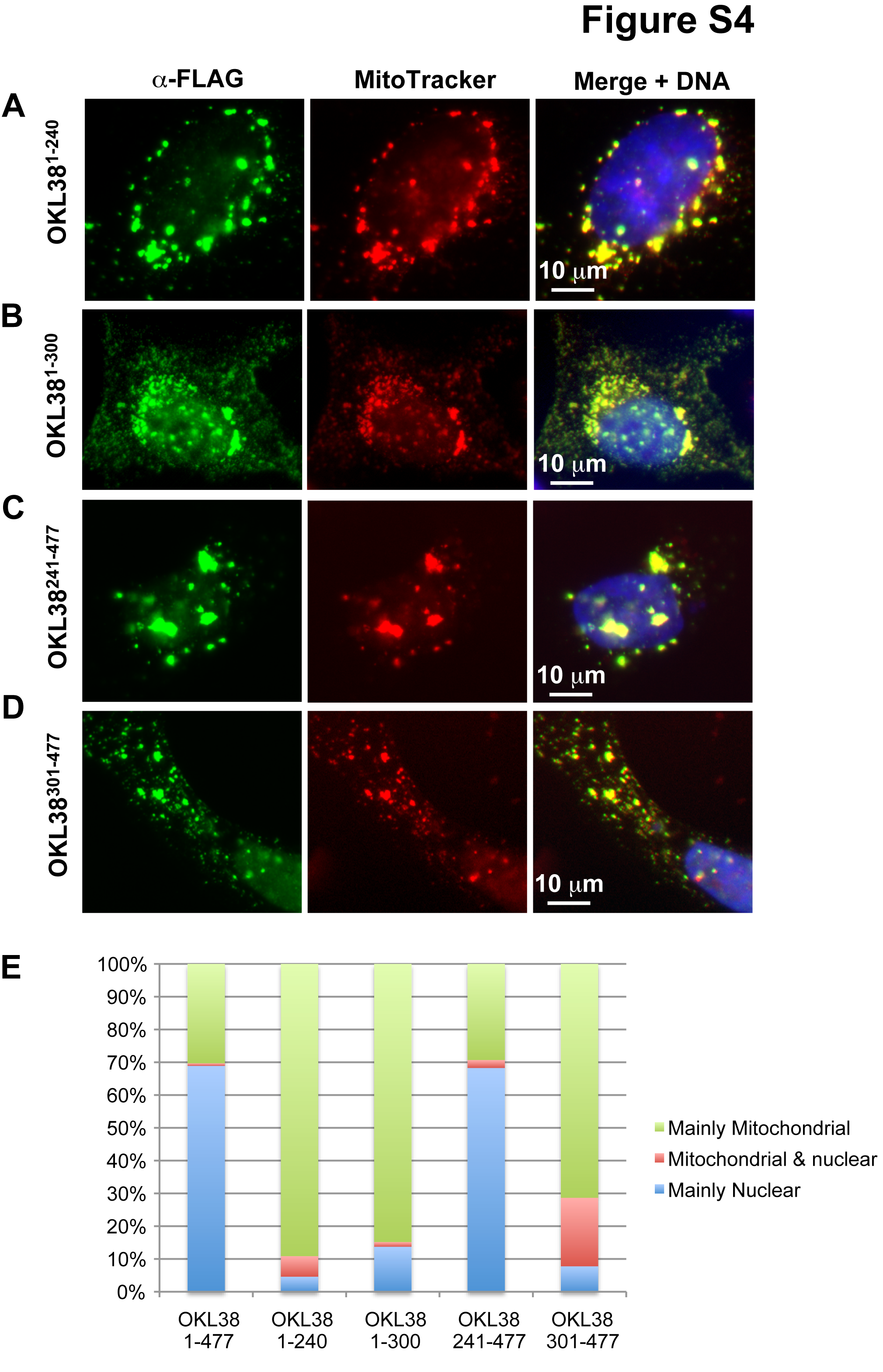

Supplement: Figure S4 — Subcellular distribution of FLAG-OKL38 truncations in U2OS cells. (A) OKL38 residues 1–240. (B) OKL38 residues 1–300. (C) OKL38 residues 241–477. (D) OKL38 residues 301–477. (E) Over 250 cells from independent experiments were scored by two observers for subcellular localization of OKL38 and its truncation derivatives. Percentages of cells with mainly mitochondrial, mainly nuclear, or both mitochondrial and nuclear OKL38 localization are shown in the bar graphs. (TIF) [file pone.0043362.s004.tif]

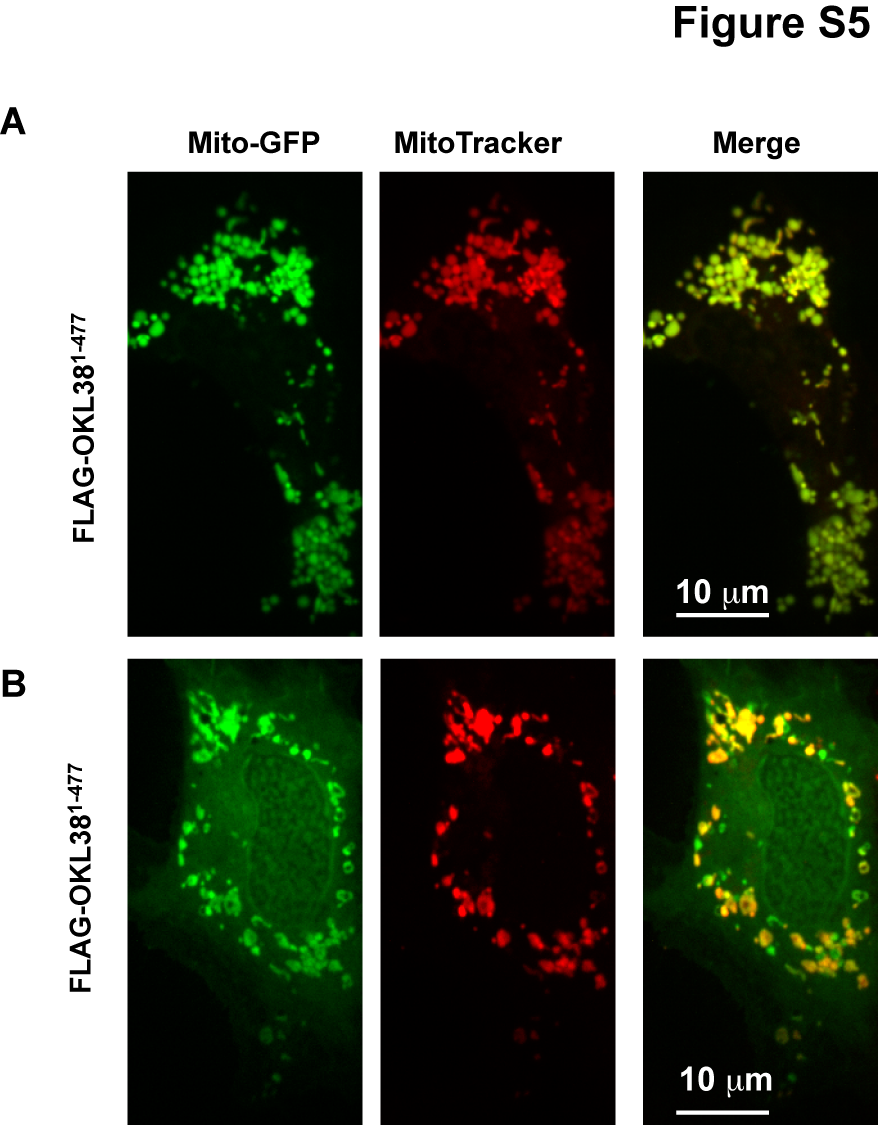

Supplement: Figure S5 — Effects of FLAG-OKL38 on the GFP-mito reporter distribution. (A–B) FLAG-OKL38 full length construct was co-transfected with the GFP-mito reporter construct in U2OS cells. Fragmentation of mitochondria and formation of large mitochondria speckles were detected in live cell imaging analyses. (TIF) [file pone.0043362.s005.tif]

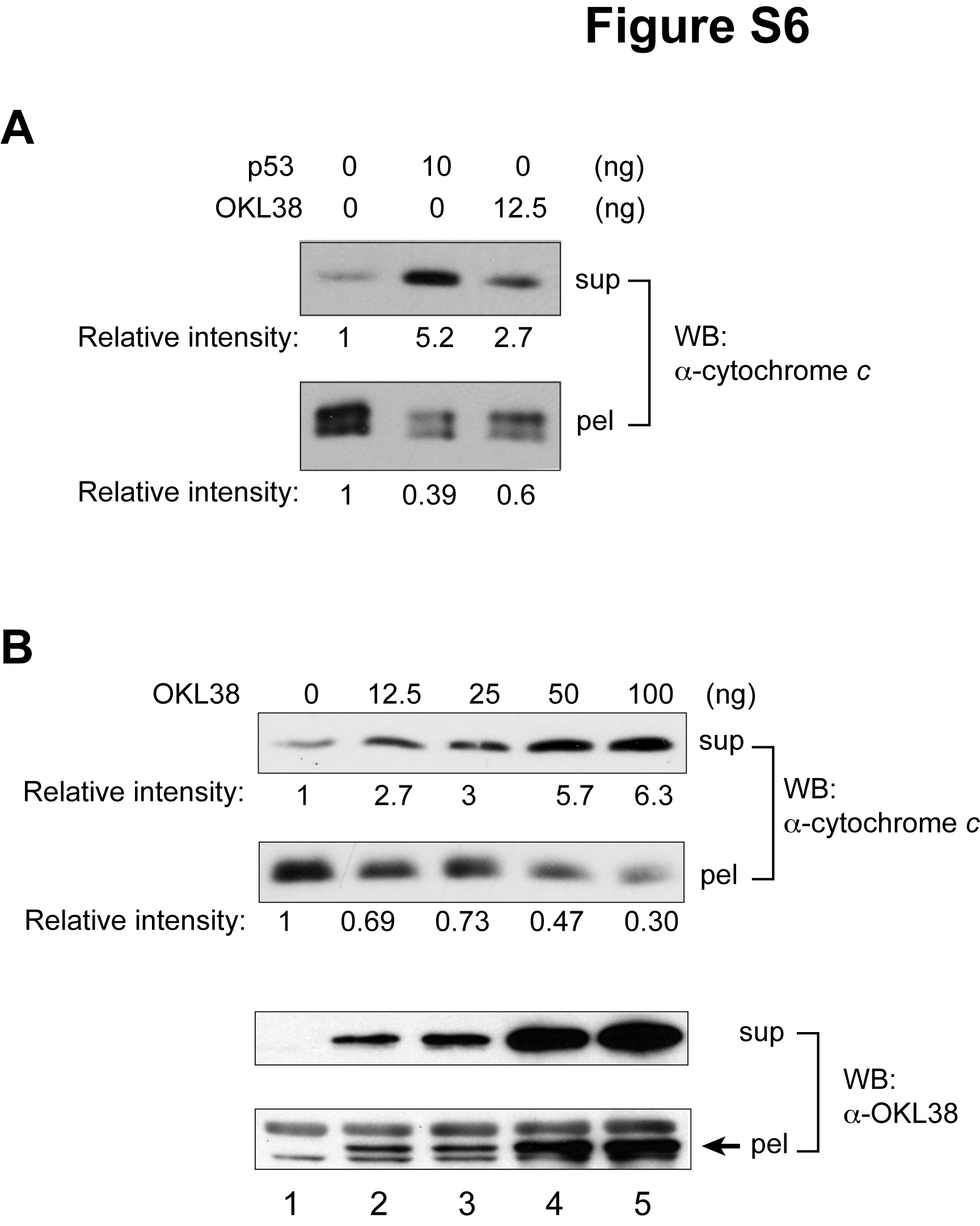

Supplement: Figure S6 — Relative abundance of cytochrome c in pellet and supernatant after p53 or OKL38 incubation. (A) The amount of cytochrome c in the supernatant (upper panel) or in the pellet (lower panel) was detected by Western blot. The relative signal under each treatment condition was measured using the NIH Image J program. With the increased amount of cytochrome c released, a concomitant decrease of cytochrome c from the pellet was detected. (B) The effect of OKL38 on mitochondrial cytochrome c release was tested using an isotonic buffer containing 125 mM KCl and other salts. The amount of cytochrome c in the supernatant or in the pellet (two upper panels) was detected by Western blot. The relative signal was measured using the NIH Image J program. With the increased amount of OKL38, a concomitant increase of cytochrome c release was detected. The amount of OKL38 in the supernatant or the pellet (two lower panels) was also monitored by Western blot. Arrow denotes the recombinant OKL38 detected in the mitochondrial pellet. (TIF) [file pone.0043362.s006.tif]
